# A Miniaturized Quad-Stopband Frequency Selective Surface with Convoluted and Interdigitated Stripe Based on Equivalent Circuit Model Analysis

**DOI:** 10.3390/mi12091027

**Published:** 2021-08-27

**Authors:** Jian Dong, Yan Ma, Zhuangzhuang Li, Jinjun Mo

**Affiliations:** 1 School of Computer Science and Engineering, Central South University, Changsha 410075, China; dongjian@csu.edu.cn (J.D.); my990906@csu.edu.cn (Y.M.); lizhuangzhuang@csu.edu.cn (Z.L.); 2 School of Information and Communication, Guilin University of Electronic Technology, Guilin 541004, China

**Keywords:** frequency selective surface, quad-stopband, miniaturization, angle and polarization stability

## Abstract

This paper presents a miniaturized frequency selective surface (FSS) based on the convoluted and interdigitated stripe with multiple narrow passbands/wide stopbands in the L-/S-/C-/X-/Ku-/K-band. By using the convoluted and interdigitated stripe, the coupling inside is well controlled, so that the spatial efficiency is maximized to provide a high miniaturization. An equivalent circuit model is presented to reveal the working mechanism of the proposed FSS. The proposed structure forms four transmission band rejections of 3 dB in 1–6.65 GHz, 8.35–16.9 GHz, 18.0–24 GHz, and 24.50–27.84 GHz. The size of the unit cell is 0.09λ_0_ × 0.09λ_0_, where λ_0_ is the wavelength of the first resonance frequency. The proposed FSS has a good angle stability and polarization stability in a scanning range up to 60°. For verification, an FSS prototype has been fabricated and measured. The measured results were in agreement with the simulated results. The proposed FSS can be used in practical applications such as radomes, antenna reflectors, and spatial filters.

## 1. Introduction

A frequency selective surface (FSS) is composed of periodically arranged patch-type units or aperture-type units and dielectric substrates, with spatial filtering characteristics. The patch-type units have a bandstop characteristic, while the aperture-type units have a bandpass characteristic [1,2,3,4]. Due to its potential values in the construction of microwave components with frequency-domain filter characteristics, FSS has been widely applied to radomes, output couplers in the infrared band, reflector antennas for frequency multiplexing, polarization converters or polarization selectors in the microwave frequency band, filters in the terahertz range, and so on. The scope of application covers almost the entire frequency band [5,6,7,8,9,10]. Due to space constraints in practical applications, a compact FSS is required to incorporate a large number of unit cells in a specific area to maintain the nature of the infinite periodic array. In addition, the FSS formed by large units easily leads to the phenomenon of grating blades [11,12]. With the rapid development of multifunctional modern communication systems, the traditional single communication system terminal cannot meet the requirements. There is a huge demand for FSS with multiband performance [13,14]. For the purpose of electromagnetic shielding, if FSS can act as a bandstop filter on as many wireless frequency bands as possible while remaining transparent to other unused frequencies, then FSS becomes more effective. As a result, the FSS composed of miniaturization and multiband unit cells has important significance [15].

The current research on multiband FSS mainly focuses on dual-band FSS. Previously, [16] proposed a low-profile dual-band FSS. The double stopband characteristic is realized by the multiresonant element square ring and crossed dipoles. Vertical vias are loaded into the crossed dipoles to increase the capacitance and inductance in the high-frequency band and reduce the corresponding resonance frequency. Although the band rejection characteristic for transverse magnetic (TM) polarization is relatively stable, for transverse electric (TE) polarization, as the incident angle increases, the first suppression band remains almost unchanged, while the second suppression band moves downward. In addition, [17] proposed a method of designing a dual-band miniaturized-element frequency selective surface (MEFSS). The proposed structure consists of grids and complementary capacitor patches arranged periodically in two dimensions, separated by a dielectric substrate. This structure is based on an inductively coupled MEFSS, which uses a capacitively loaded dielectric pad as its main resonator. The two operating frequency bands of the MEFSS correspond to the first and second resonance frequencies of the resonator. For TE and TM incident polarization, the incident angle is relatively stable in the range of 45°. In [18], an FSS with a wide passband and ultra-wide stopband performance was studied. The proposed FSS is composed of three metal layers and a cascading multilayer dielectric substrate. In the L/S frequency band, a new type of inductance layer with a spiral zigzag line shape is proposed to synthesize a large inductance to increase the passband bandwidth. When the incident angle is in the range of 0° to 45°, an ultra-wide reflection band with good and stable performance can be obtained. In [19], a thin multilayer polarization-independent FSS was proposed. The FSS includes three metal layers. One is a four-legged metal cross on the top surface, the other is on the bottom surface, and the middle layer is a Jerusalem cross. This structure reflects the bandpass characteristics of 7.15 GHz and 13.02 GHz and the bandstop response of 8.22 GHz. This structure is not sensitive to polarization. Under different incident angles, the frequency shift of the lower two frequency bands is less than 5%, while the frequency shift of the higher transmission frequency band is up to 12%. Although the tri-band FSS can achieve its operating characteristics, the filtering characteristics are singular, that is, all bandstop or all bandpass, which makes it easy to cause bandstop/bandpass of the whole frequency band. The design of the tri-band FSS can be implemented in a way that a ring structure is nested in itself. The authors of [20] mentioned a tri-band blocking FSS operating at 1.8 GHz, 3.5 GHz, and 5.5 GHz. The FSS has a single-sided FR4 substrate and a single metal layer. The metal layer is composed of a square ring, a dodecagonal ring, and a Jerusalem cross ring. Although the FSS realizes tri-band, the size of the unit is large, which is not conducive to the miniaturization of the FSS and limits the practical use. There have been similar designs proposed in [21,22,23,24,25]. The design of a multiband FSS can theoretically be realized in a multilayer cascade mode, However, the increase in the number of unit layers and the increase in response characteristics have brought difficulties to multiband design. Multilayered hyperboloid processing is prone to misalignment. Limited by manufacturing process, upper and lower parts of the cell cannot be aligned completely, which not only affects the coherence of resonance between the layers, but also increases the loss and changes the design performance.

All the above-mentioned designs use multiple metal layers, which not only requires high processing accuracy, but also increases the production cost. In the conformal design of the radome and the subreflector, the multilayer FSS has a complex structure and high sensitivity, and its transmission characteristics need to be designed. Therefore, it is necessary to design a simple, compact, and symmetrical FSS structure, which has the advantages of simple structure, low cost, good angular stability, and polarization insensitivity. Convolution and interdigitation techniques improve space efficiency by making a compact combination of conductive patterns, and these two techniques will increase the capacitance or inductance of the component, and then contribute to miniaturization. Starting from the convolution structure in [26], we present a miniaturized single-layer quad-stopband FSS in detail in this paper by describing its design concept, design evolution, equivalent circuit model analysis, polarization and angle stability analysis, measurement setup, and comparative analysis. The proposed FSS maintains polarization stability within a 60° incident angle with multiband characteristics and has practical engineering significance. The proposed FSS has an ultra-thin profile. In addition, compared with the multilayer structure, the single-layer FSS has the advantages of strong usability, low cost, light weight, easy manufacturing, and insensitive response.

The remainder of this paper is organized as follows. Section 2 introduces the design concept and describes the proposed unit cell design, and gives the specific parameters of the unit cell. Both the equivalent circuit model analysis of the proposed FSS and its performance analysis under different conditions, including structural parameter changes, different polarization modes, and different incident angles are given in Section 3. Section 4 presents a comparison of measured and simulated results. To emphasize the advantages of the proposed FSS over similar structures previously implemented, a comparative study was conducted. Finally, Section 5 provides concluding remarks.

## 2. Design of the FSS

### 2.1. Design Concept

When FSS is irradiated by the incident wave, a unit cell of FSS can be regarded as a resonant circuit. An *L*-*C* parallel resonance is the basic principle of bandpass FSS, where *L* and *C* represent the equivalent inductance and capacitance of the unit cell, respectively. When designing conductive patterns, both space efficiency and convolution principles must be considered. The former determines the total length of the stripes, and the latter can provide an appropriate balance between the capacitive part and the inductive part.

For bandpass FSS, the capacitors distributed in the FSS unit can be divided into intercapacitor (*C*_inter_) and intracapacitor (*C*_intra_), where *C*_inter_ is the capacitance inside the element and *C*_intra_ is the capacitance between the elements. *C*_intra_ will introduce a new high-frequency resonance at high frequencies, while *C*_inter_ will increase the equivalent inductance (*L*_E_) at low frequencies. The main resonance frequency (*F*) is determined by the following formulas.

When $0<\omega \leq \sqrt{1/LC_{\mathrm{intra}}}$

(1) $$\frac{1/(j\omega C_{\mathrm{intra}})\times j\omega L}{1/(j\omega C_{\mathrm{intra}})+j\omega L}=j\omega L_{E}$$

(2) $$L_{E}=L/\left(1-\omega^{2}LC_{\mathrm{intra}}\right)$$

(3) $$F=1/2\pi \sqrt{L_{E}C_{\mathrm{inter}}}$$

Therefore, reducing the resonance frequency is equivalent to increasing the inductance or capacitance of the unit cell. For a square slot unit cell, resonance will occur when the perimeter of the square slot is approximately an integer multiple of the incident wavelength. Therefore, we can increase its physical size in a limited area by bending the square slot. In other word, we can reduce the resonance frequency without increasing the area of the unit cell and achieve the characteristics of miniaturization.

### 2.2. Design Evolution and Configuration

**Figure 1:**
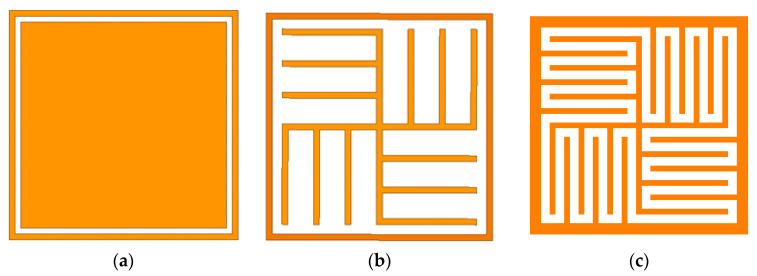
Design flowchart: (**a**) initial structure; (**b**) convoluted square stripes; (**c**) added branches to be convoluted and interdigitated stripe.

The design flow chart is shown as in Figure 1. The design process can be divided into four steps. The first step is the selection of the initial structural model. We choose the square gap ring element as the initial model (see Figure 1a). In the second step, in order to achieve miniaturization, we convolute the square stripe in the unit cell (see Figure 1b). To further achieve miniaturization, we add branches to the outermost square ring patch for interdigital processing (see Figure 1c). Finally, we carry out specific parameter optimization.

**Figure 2:**
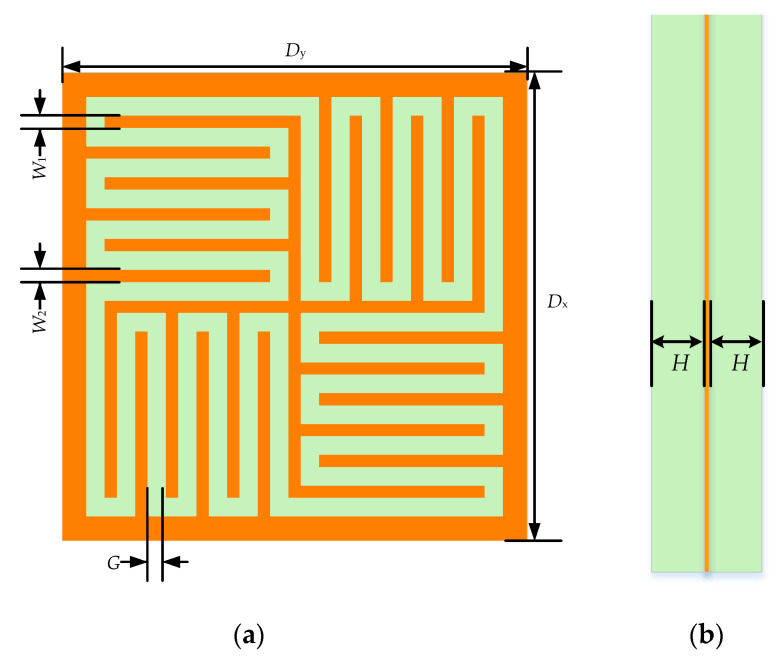
Diagrammatic sketch of the proposed unit: (**a**) top view; (**b**) side view.

The proposed FSS unit cell is shown in Figure 2. This compact convoluted and interdigitated arrangement effectively increases the corresponding equivalent inductance. By adjusting the distance of the slot, the corresponding equivalent capacitance can be increased. Therefore, by using convolution and interdigitation technologies, the internal coupling is well controlled, the phenomenon of grating lobes is avoided, and the space efficiency and miniaturization are improved to the maximum extent. In addition, this centrosymmetric structure improves the stability of FSS under different polarizations and incident angles and ensures the practicability of FSS.

**Figure 3:**
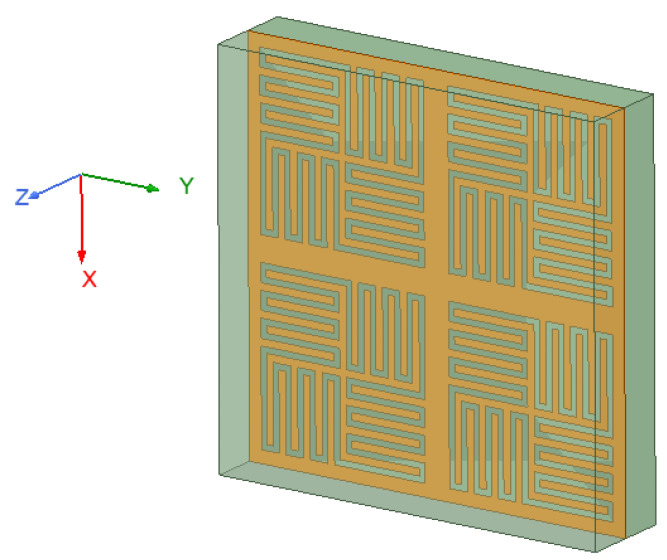
Perspective of overall structure of the proposed unit.

The proposed FSS with 2 × 2 array elements is shown in Figure 3, in which a metal layer is sandwiched by two dielectric layers. The array is arranged in a square grid. In the figure, *D*_x_ and *D*_y_ are the periods of the proposed FSS in the *x*-axis and *y*-axis directions, and their values can be equal or unequal, but the values must meet the FSS requirements for array spacing to avoid or delay the appearance of grating lobes. To make the structure highly symmetrical and enhance the angular and polarization stability of the new structure, *D*_x_ = *D*_y_ is taken here. *W*_1_ and *W*_2_ are the widths of the inner and outer branches of the square ring aperture. Also, *W*_1_ = *W*_2_ is taken here. *G* is the width of the aperture. To be able to insert more branches, the value of *G* should be as small as possible. However, the value must not be too small, otherwise, the electromagnetic wave will no longer propagate along with the curved aperture.

The model consists of a double-layer dielectric material and a single-layer metal surface, where the metal layer is located in the center of the dielectric layer. The green part is the dielectric substrate. The orange part is the metal, and its thickness can be ignored in the simulation process. The material of the top and the bottom dielectric layer is Rogers RT 5880 (tm) with $\varepsilon_{r}=2.2$, tanδ=0.0009, and H=0.8mm. The proposed FSS has a miniaturized size of only 3.94 mm × 3.94 mm (0.09λ × 0.09λ, where λ is the free space wavelength at 7.5 GHz). Through analysis and optimization by HFSS software, the specific structural parameters are listed in Table 1.

## 3. FSS Analysis

### 3.1. Equivalent Circuit Model Analysis

**Figure 4:**
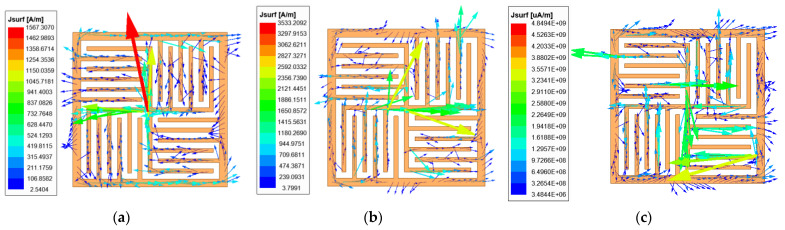
Surface current diagram at: (**a**) *f*_1_ = 7.3 GHz, (**b**) *f*_2_ = 17.1 GHz, (**c**) *f*_3_ = 23.9 GHz.

To better understand the operation principle, we simulated the surface current and surface electric field to construct the equivalent circuit model. Figure 4 shows the distribution of surface current at normal incidence at frequencies of 7.3 GHz, 17.1 GHz, and 23.9 GHz. It can be seen from Figure 4 that at 7.3 GHz, the currents are mainly distributed around the outer branch and the central cross branch; at 17.1 GHz, the currents are mainly distributed around the central cross branch; and at 23.9 GHz, the currents are mainly distributed around the other small branch. According to observations, due to the branch structure design, three different resonance paths are provided. Then, four stopbands will be produced. The results show that the short arm with a smaller electrical length helps to produce higher resonance, and the increase of electrical length helps to produce lower resonance.

**Figure 5:**
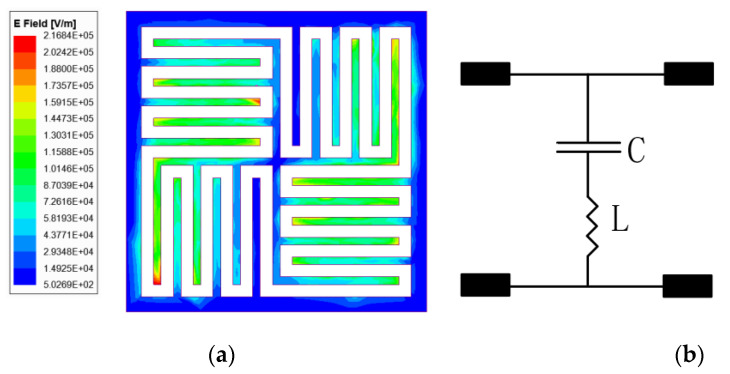
Resonance of the stripe at 7.3 GHz: (**a**) schematic diagram of surface electric field (**b**) equivalent circuit model diagram.

When the proposed FSS structure is illuminated by the 7.3 GHz incident wave, the surface electric field and surface current appear as shown in Figure 4a and Figure 5a. Near the outer branch and the central cross branch, the surface electric field is weak and the surface current is strong. However, on the other small branches, the surface electric field is strong and the surface current is weak, so the equivalent circuit of this structure at 7.3 GHz could be modeled by a series *L*-*C* resonator which is shown in Figure 5b. *C* indicates the capacitance in the central zone of an element and *L* is the inductance of a whole convoluted and interdigitated stripes.

**Figure 6:**
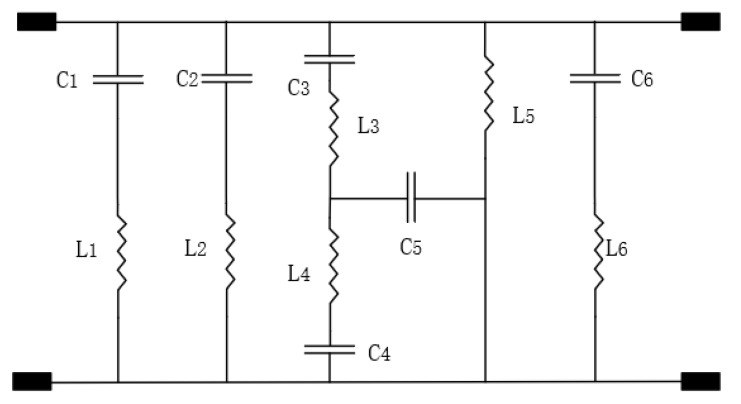
Equivalent circuit model diagram (*C*_1_ = 0.0196 pF, *C*_2_ = 0.109 pF, *C*_3_ = 0.0107 pF, *C*_4_ = 0.0314 pF, *C*_5_ = 0.0274 pF, *C*_6_ = 0.0045 pF, *L*_1_ = 3.275 nH, *L*_2_ = 2.255 nH, *L*_3_ = 2 nH, *L*_4_ = 2.26 nH, *L*_5_ = 1.5675 nH, *L*_6_ = 6.465 nH).

**Figure 7:**
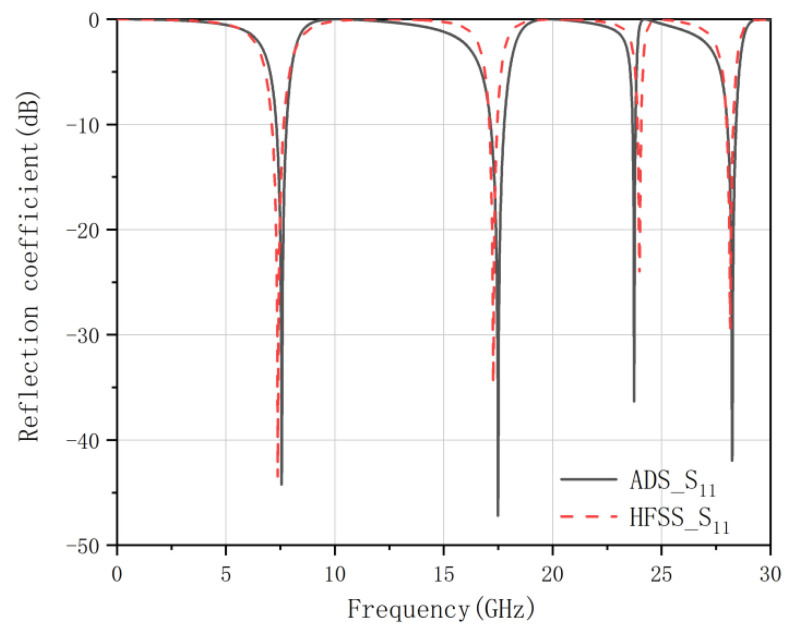
Comparison of reflection response of the proposed FSS calculated by HFSS and ADS.

With the method above, the equivalent circuit model of the proposed FSS structure can be obtained, as shown in Figure 6. The effectiveness of the equivalent circuit model is proved by the performance comparison between the reflection coefficient calculated by ADS and HFSS. It can be observed in Figure 7 that both curves fit well in the range of 0–30 GHz.

### 3.2. Parametric Variation

**Figure 8:**
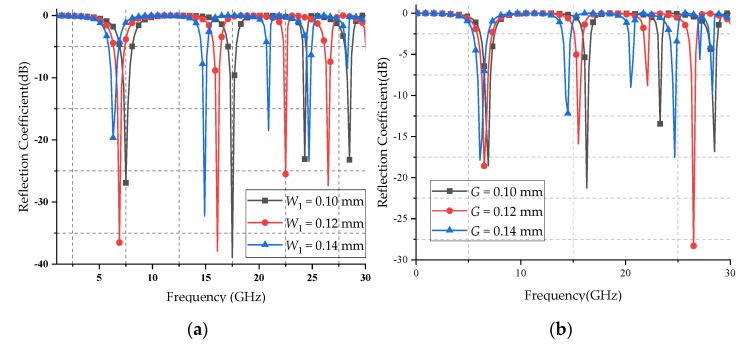
Reflection coefficients of the proposed FSS with different values for (**a**) parameter W_1_; (**b**) parameter *G*.

Selecting reasonable cell structure parameters can control the distribution of the induced current, thereby obtaining the required transmission characteristics. The unit structure parameters not only affect the operating frequency of the FSS, but also affect the operating bandwidth, insertion loss, polarization stability, and oblique incidence stability. Therefore, it is necessary to examine the influence of the changes of *G* and *W*_1_ parameters on the frequency response characteristics of the proposed FSS. Figure 8a,b shows the reflection coefficient curves with different parameter values for *W*_1_ and *G*, respectively. Table 2 counts the 3 dB bandwidth of stopbands with different *W*_1_ and *G*.

Figure 8 shows that the proposed FSS has flat top characteristics in the stopband without significant fluctuations, indicating a high degree of stability in the stopband. Also, there are steep edges on both sides of the stopband, indicating that it has good frequency selective characteristics. As shown in Table 2, with the increase of *G* and *W*_1_, the four frequency bands shift towards the low frequency direction as a whole, but the shift of the high frequency band is greater. When *W*_1_ and *G* have the same amount of change, the change of the *W*_1_ value has more effect on the transmission characteristic curve. Therefore, *W*_1_ can be adjusted to determine the general position of the stopband, and *G* can be adjusted to determine the precise position of the stopband. In actual application scenarios, by reasonably adjusting *W*_1_ and *G* of the unit cell, multiband requirements can be satisfied.

### 3.3. Polarization Stability

When the FSS works, the effect of the polarization mode and incident angle of the electromagnetic wave incident on the FSS is unknown. If FSS shows different transmission characteristics and reflection characteristics for TE-polarized wave and TM-polarized wave, FSS may not work unstably. To eliminate the influence of the polarization of the incident wave on the performance of the FSS, the designed FSS must have similar frequency response characteristics for the TM wave and the TE wave.

**Figure 9:**
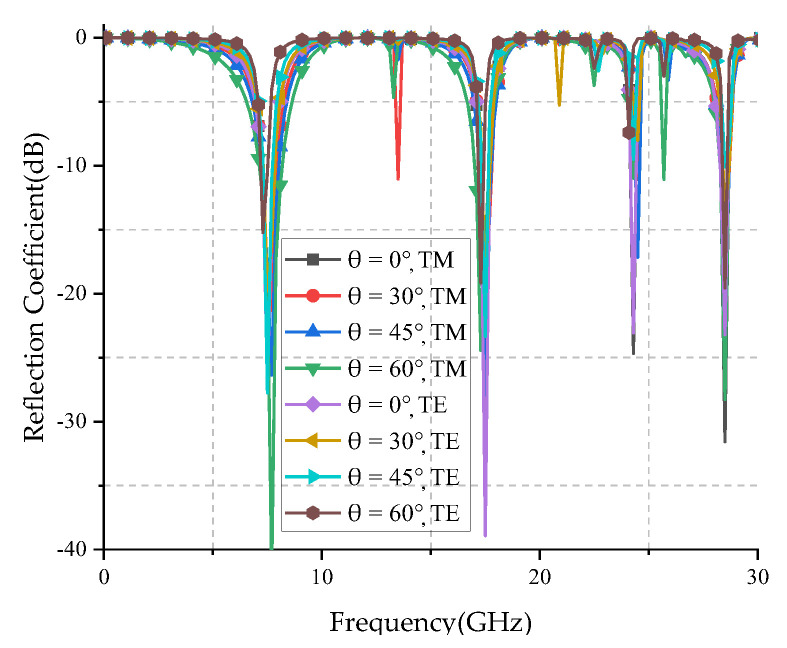
Reflection coefficients for various oblique angles of incidence under TE- and TM-polarized waves.

Figure 9 shows the reflection coefficient curves for various oblique angles of incidence under TE- and TM-polarized waves. It can be seen from the figure that the proposed FSS has multiple resonance points within 30 GHz, and has multiband characteristics. Table 3 shows the resonant frequency and the deviation rate of the proposed FSS with different polarized obliquely incident waves at different angles. Here, the parameter of average polarization shift is defined to analyze the polarization stability of the proposed FSS and calculated as follows:

(4) $$\bar{\Delta f_{a}}=\frac{\sum_{1}^{n}\frac{\left|f_{a}^{\mathrm{TM}}-f_{a}^{\mathrm{TE}}\right|}{f_{a}^{\mathrm{TM}}}}{n}\times 100\%$$

where $f_{a}^{\mathrm{TE}}$ is the resonant frequency of the TE wave incidents at θ angle, $f_{a}^{\mathrm{TM}}$ is the resonant frequency of the TM wave incidents at θ angle, *n* is the total number of θ angle. The lower the value, the better the polarization stability.

From Table 3, it can be seen that the FSS has an offset of 2.60% at the first resonance frequency (*f*_1_), no frequency offset at the second resonance frequency (*f*_2_), and only 0.21% at the third resonance frequency (*f*_3_). This shows that the polarization stability of the FSS is very good.

### 3.4. Angle Stability

Due to the uncertainty of the direction of the electromagnetic wave, the FSS, if sensitive to the incident angle, will have a large deviation at its resonant frequency when it resonates. If the deviation is too large, the FSS cannot guarantee to keep the proper spatial filtering function within the working frequency band. Therefore, the resonant frequency of the designed FSS must have good angle stability under different polarized oblique incidences to ensure that the FSS can achieve stable operation for a fixed frequency band.

**Figure 10:**
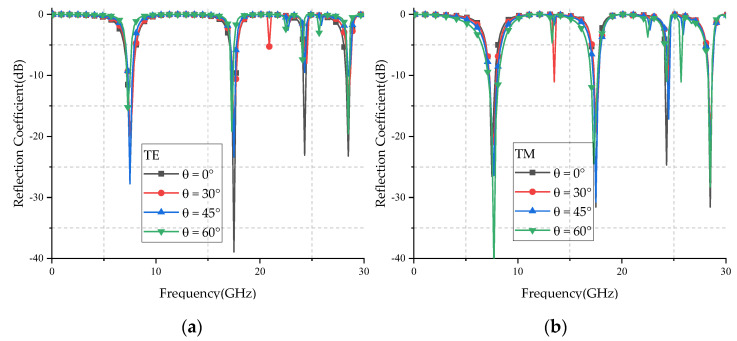
Simulated reflection coefficients for various oblique angles of incidence: (**a**) TE polarization; (**b**) TM polarization.

Figure 10 shows the simulated reflection coefficients for various oblique angles of incidence. Here, the angle mean deviation is defined to analyze the angle stability of the proposed FSS and calculated as follows:

(5) $$\delta f_{p}=\frac{\left|f_{p}^{\mathrm{normal}}-f_{p}^{a_{1}}\right|+\left|f_{p}^{\mathrm{normal}}-f_{p}^{a_{2}}\right|+\cdots +\left|f_{p}^{\mathrm{normal}}-f_{p}^{a_{n}}\right|}{nf_{p}^{\mathrm{normal}}}\times 100\%$$

where $f_{p}^{\mathrm{normal}}$ is the resonant frequency at normal incidence; $f_{p}^{a_{i}}$ is the resonant frequency at $a_{i}$ angle; *n* is the total number of oblique incident angles. The lower the value, the better the angle stability.

Table 3 counts the three resonant frequencies and $\delta f_{p}$ when TE and TM waves are incident on the proposed FSS at different angles. It can be seen from Table 3 that the average angle deviation of the proposed FSS does not exceed 1% when the TE wave is incident, and the average angle deviation does not exceed 3% when the TM wave is incident. All the results show the proposed FSS has high angle stability characteristics.

## 4. Results and Discussion

**Figure 11:**
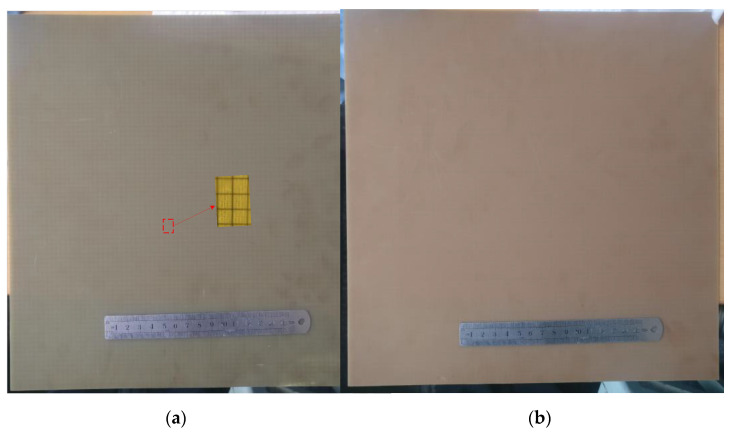
Fabricated prototype of the proposed FSS: (**a**) front view; (**b**) back view.

To verify that the proposed FSS is effective in practical applications, the proposed structure was fabricated and measured. Figure 11 shows the fabricated prototype of the proposed quad-stopband miniaturized FSS. The prototype had a size of 315.2 mm × 315.2 mm and contained 80 × 80 units. The metal layer was sandwiched between two dielectric substrates. The medium bonding the two dielectric layers was FR4, and its thickness was 0.075 mm.

**Figure 12:**
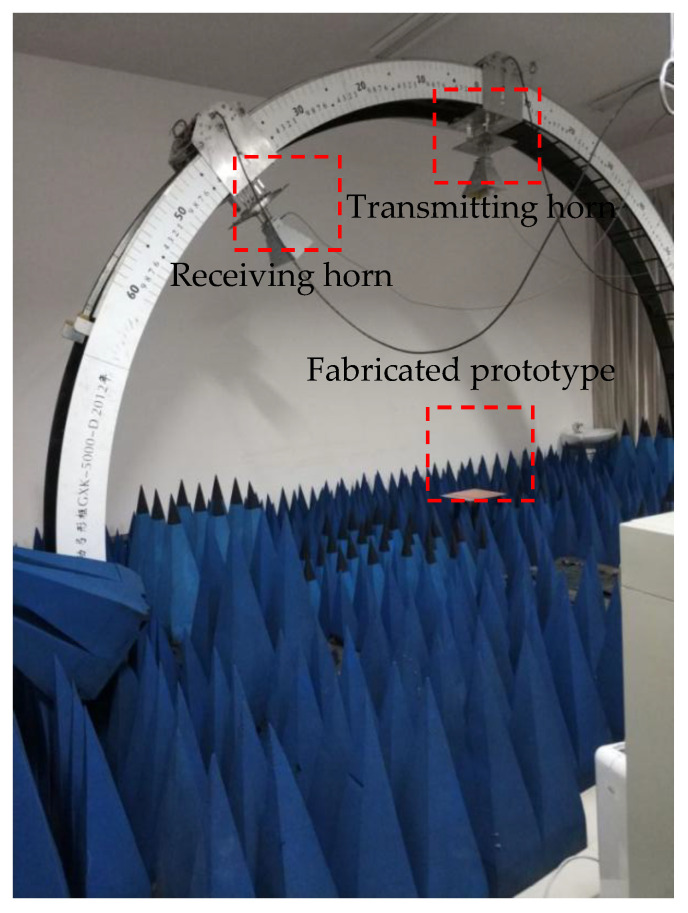
Measurement setup.

The FSS experimental test system is shown in Figure 12. The test system mainly included a vector network analyzer and two wideband horn antennas for transmitting and receiving electromagnetic waves. The test was carried out in two steps. Firstly, we used a metal plate to calibrate the reflection coefficient. Theoretically, the electromagnetic waves emitted by the emitting horn can be reflected without any loss when there is no shielded surface, but in actual measurement, loss will inevitably be caused by factors such as devices. So, calibration is necessary for the measurement when there is no FSS. Through the measurement, the value of its loss can be obtained, which can be used to reduce the error of subsequent results. Secondly, we replaced the metal plate with the fabricated prototype of the proposed FSS and repeated the above measurement. All received signals were processed by a vector network analyzer, and the required reflection coefficient value could be obtained. Using the result of the second measurement and removing the error caused by the instrument itself, the frequency domain response curve of the measured sample could be obtained.

**Figure 13:**
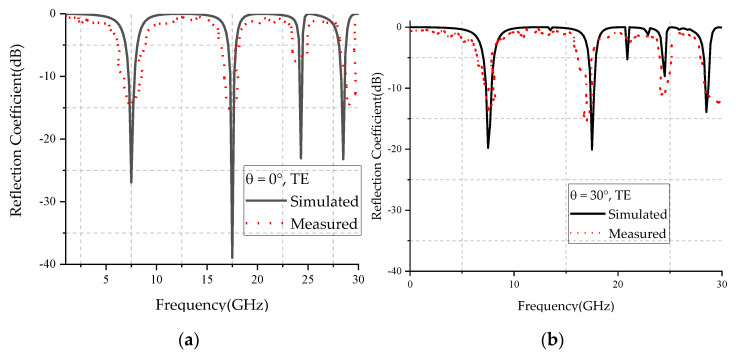

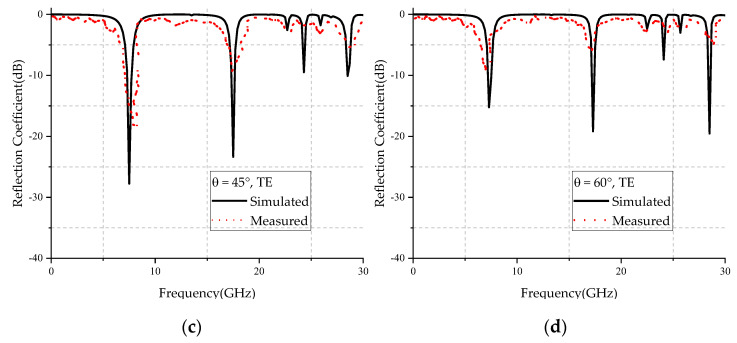
Comparison of simulated and measured results of TE wave incident at: (**a**) 0 degrees, (**b**) 30 degrees, (**c**) 45 degrees, and (**d**) 60 degrees.

**Figure 14:**
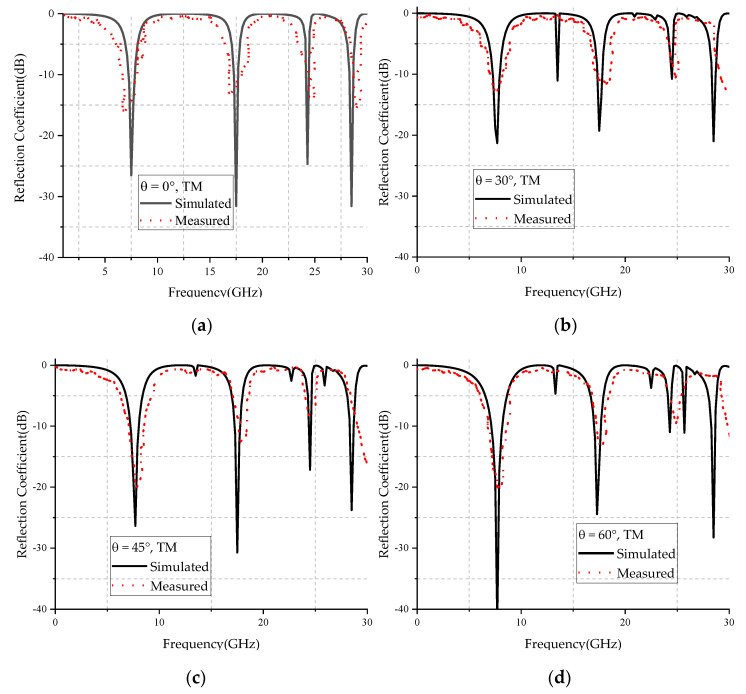
Comparison of simulated and measured results of TM wave incidence at: (**a**) 0 degrees, (**b**) 30 degrees, (**c**) 45 degrees, (**d**) 60 degrees.

Figure 13 and Figure 14 show the simulated and measured reflection coefficient curves for TE and TM polarizations in different incidence angles, respectively. From the figures, we can see that the simulated results were basically in agreement with the measured results, and the center frequency of different polarized waves did not change with the increase of the incident angle. The difference between simulated and measured curves may have been caused by the errors of fabrication and measurement. The ripples of the measured curve may have been caused by the multipath interference in the testing environment.

Table 4 compares the proposed FSS with other work given in [11,17,21,27,28,29,30,31,32,33,34,35], considering the aspects of unit cell size, operation band, number of metal layers, number of substrate layers, operating frequency band, and range of incident angles. As seen in Table 4, the proposed FSS has a more compact size than structures designed in [11,21,27,28,29,30,31,32,33,34,35]. Moreover, compared with [17,29,32,35], the proposed FSS structure has more operating frequency bands. Also, this structure has a larger range of incident angles than [17,21,28,32,35].

## 5. Conclusions

In this paper, a miniaturized quad-stopband FSS was designed based on convoluted and interdigitated stripe. The equivalent circuit model was analyzed, and simulation verification and experimental testing were performed. Also of note, it can be easily manufactured by using low-cost PCB technology. It turns out that for TE and TM modes, the response is not sensitive to the incident angle. The structure shows a very stable resonance frequency with an incident angle of up to 60°.The measured results are basically consistent with the simulated results. The results show that the proposed FSS achieves miniaturization and quad-band characteristics, and it has more frequency band choices than the traditional dual band FSS.

The proposed FSS forms four transmission band rejections of 3 dB in 1–6.65 GHz, 8.35–16.9 GHz, 18.0–24 GHz, and 24.50–27.84 GHz. The center frequencies of the passband are 7.5 GHz, 17.5 GHz, and 24.3 GHz, respectively. According to the working characteristics of the passband and stopband, the proposed structure can be applied to the design of the secondary reflector, reflecting the signal in the stopband and transmitting the signal in the passband. This multiband multiplexing reflector antenna has potential applications in satellite communications, navigation, and deep space exploration. The second option is to design a radome for radar applications to realize the free transmission of its own signal and the stealth of the enemy signal.

## Figures and Tables

**Table 1:** Parameter values shown in Figure 2.

Parameter	*H*	*G*	*W*_1_	*W*_2_	*D*_x_	*D*_y_
**Value (mm)**	0.80	0.16	0.10	0.10	3.94	3.94

**Table 2:** The 3 dB bandwidths of stopbands with varying *W*_1_ and *G*.

Parameters (mm)	3dB Bandwidth (GHz)			
	1st Band	2nd Band	3rd Band	4th Band
*W*_1_ = 0.10	1.00–6.65	8.35–16.90	18.04–24.00	24.50–27.84
*W*_1_ = 0.12	1.00–6.10	7.62–15.56	16.55–22.21	22.69–25.96
*W*_1_ = 0.14	1.00–5.65	7.05–14.43	15.28–20.60	21.08–24.26
*G* = 0.10	1.00–6.19	7.42–15.93	16.67–23.10	23.51–27.94
*G* = 0.12	1.00–5.94	7.20–15.14	15.91–21.76	22.25–26.01
*G* = 0.14	1.00–5.52	6.81–14.02	14.75–20.31	20.82–24.23

**Table 3:** The resonance frequency and offset of the proposed FSS for different waves incident modes at different angles.

Modes	TE (GHz)				TM (GHz)				$\bar{\Delta f_{a}}(\%)$	$\delta f_{p}^{\mathrm{TE}}(\%)$	$\delta f_{p}^{\mathrm{TM}}(\%)$
Angle (deg.)	0	30	45	60	0	30	45	60			
*f*_1_	7.5	7.5	7.5	7.3	7.5	7.7	7.7	7.7	2.60	0.89	2.67
*f*_2_	17.5	17.5	17.5	17.3	17.5	17.5	17.5	17.3	0	0.38	0.38
*f*_3_	24.3	24.5	24.3	24.1	24.3	24.5	24.3	24.3	0.21	0.55	0.27

**Table 4:** Comparative analysis of the proposed FSS and other reported FSSs.

Ref.	Unit Cell Size (mm)	No. of Stopbands	No. of Metal Layers	No. of Substrate Layers	Operating Frequency Band	Angle Stability (deg.)
[11]	6.6 × 6.6 × 1	2	1	1	L, S, C, X	60
[17]	1.9 × 1.9 × 4	2	5	4	Ku, Ka	45
[21]	50 × 50× 1	3	1	1	GSM	45
[27]	16 × 16 × 1.6	2	1	1	WiMAX, WLAN	60
[28]	46 × 46 × 1	3	1	1	GSM	45
[29]	6 × 6× 11.5	1	2	5	Ku	60
[30]	5 × 5× 0.508	3	1	1	X, Ku, Ka	80/80/60
[31]	4 × 4× 1	2	1	1	Ku, Ka	60
[32]	10 × 10 × 1.6	3	2	2	WiMAX, WLAN, X	50
[33]	8.4 × 8.4 × 0.8	2	1	1	L, S	60
[34]	8.8 × 8.8 × 0.762	2	1	1	X	60
[35]	4.0 × 4.0 × 8.78	1	4	9	L, S, C, X, Ku	30
Proposed	3.94 × 3.94 × 1.6	4	1	2	L, S, C, X, Ku, K, Ka	60

## Data Availability

Data are contained within the article.
